# Origin and pathophysiology of protein carbonylation, nitration and chlorination in age-related brain diseases and aging

**DOI:** 10.18632/aging.101450

**Published:** 2018-05-17

**Authors:** Efstathios S. Gonos, Marianna Kapetanou, Jolanta Sereikaite, Grzegorz Bartosz, Katarzyna Naparło, Michalina Grzesik, Izabela Sadowska-Bartosz

**Affiliations:** 1 National Hellenic Research Foundation, Institute of Biology, Medicinal Chemistry and Biotechnology, 11635 Athens, Greece; 2 Department of Biochemistry and Molecular Biology, Faculty of Biology, University of Athens, 15701 Athens, Greece; 3 Department of Chemistry and Bioengineering, Faculty of Fundamental Sciences, Vilnius Gediminas Technical University, 2040 Vilnius, Lithuania; 4 Department of Molecular Biophysics, Faculty of Biology and Environmental Protection, University of Lodz, 90-236 Lodz, Poland; 5 Department of Analytical Biochemistry, Faculty of Biology and Agriculture, University of Rzeszow, 35-601 Rzeszow, Poland

**Keywords:** oxidative stress, carbonylation, nitration, chlorination, proteasome

## Abstract

Non-enzymatic protein modifications occur inevitably in all living systems. Products of such modifications accumulate during aging of cells and organisms and may contribute to their age-related functional deterioration. This review presents the formation of irreversible protein modifications such as carbonylation, nitration and chlorination, modifications by 4-hydroxynonenal, removal of modified proteins and accumulation of these protein modifications during aging of humans and model organisms, and their enhanced accumulation in age-related brain diseases.

## Introduction

Aging, an inevitable part of the life process, is characterized by a progressive decline in physiological functions that ultimately leads to morbidity and mortality. Aging increases susceptibility to certain class of diseases. Age-related diseases constitute a considerable socioeconomic burden for contemporary societies. As human mean lifespan increases, growing incidence of these diseases has features of a pandemic. The number of people aged 65 or older is projected to grow from an estimated 524 million in 2010 to almost 1.5 billion in 2050, mostly in underdeveloped and developing countries [1]. These trends have obvious serious social and economic implications, such as healthcare costs [2].

Despite extensive studies, the molecular basis of physiological aging is still poorly understood. Reactive oxygen species (ROS), reactive nitrogen species (RNS) as well as reactive halogen species (RXS) species are believed to play a key role in the aging process. They are generated during aerobic metabolism in living organisms. The term “*reactive oxygen species*” includes both free radicals [molecules having an odd electron, like superoxide radical anion (O_2_^•-^) and hydroxyl radical (HO^•^)] and species that are not free radicals, like hydrogen peroxide (H_2_O_2_), singlet oxygen (^1^O_2_) and ozone (O_3_). The primary source of RNS is usually the nitric oxide radical (^•^NO). In consequence of ROS and RNS reactions, peroxynitrite ONOO^-^, anion of peroxynitrous acid ONOOH, may be formed via the near diffusion-limited reaction of ^•^NO and O_2_^•-^. The term “*reactive nitrogen species*” includes also nitrous acid (HNO_2_), dinitrogen trioxide (N_2_O_3_), nitrosyl anion (NO^-^), nitrosyl cation (NO^+^), nitrogen dioxide radical (NO_2_), peroxynitrate (ONOOO^-^), peroxynitric acid (ONOOOH), nitryl chloride (NO_2_Cl), and nitronium cation (NO_2_^+^) [3,4]. *"Reactive halogen species"* include HOCl, HOBr, HOI, chlorine, bromine, iodine etc. Hypohalogenous acids (HOX; X = F, Cl, Br, or I) are formed in the body mainly by oxidation of halogen ions by myeloperoxidase. The imbalance between ROS, RNS and RXS production and the antioxidant defense, in favor of prooxidants, is causes oxidative, nitr(os)ative and halogenative stress (OS, NS, XS), respectively. Although at physiological concentrations ROS, RNS and RXS can function as signaling molecules regulating cell proliferation, growth, differentiation and apoptosis [5,6] they react with and damage all classes of endogenous macromolecules including proteins, nucleic acids, lipids and carbohydrates [7]. Proteins are the main targets for such modifications as they are the most abundant cell components in the terms of mass content. The level of protein damage increases under stress conditions and can be in principle an integrative measure of the exposure to OS, NS and XS. However, protein turnover complicates this issue, the more that modified proteins in most cases are subject to preferential degradation [8]; see Chapter “Removal of modified proteins”.

**Figure 1:**
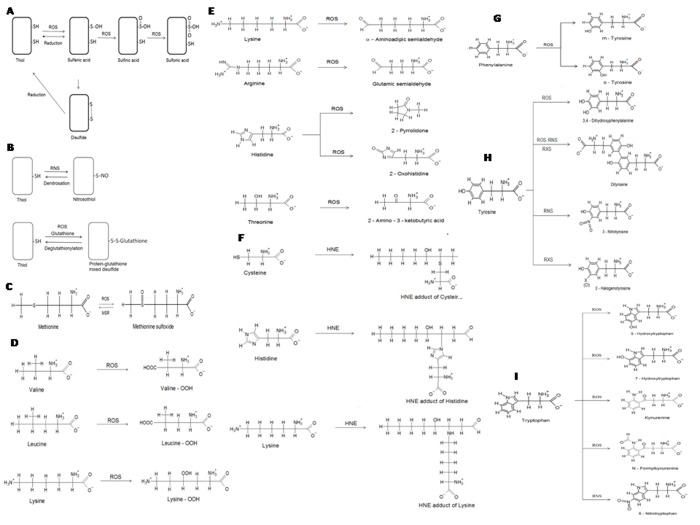
**Selected non-enzymatic protein modifications.** (**A**) oxidation of cysteine residues in proteins. Cysteine residues may be oxidized to sulfenic, sulfinic and sulfonic derivatives or form disulfide bonds. Oxidation to sulfenic acid and formation of disulfides is reversible; (**B**) modifications of cysteine residues in proteins: formation of nitrosocysteine and *S*-glutathionylation; (**C**) oxidation of methionine forms methionine sulfoxide, which may be reduced back to methionine by methionine sulfoxide reductases (MSR); (**D**) formation of hydroperoxides of valine, lysine and leucine; (**E**) formation of carbonyl derivatives of lysine, arginine, His and threonine; (**F**) formation of 4-hydroxynonenal adducts of cysteine, His and lysine; (**G**) oxidative modifications of phenylalanine; (**H**) modifications of tyrosine; (**I**) modifications of tryptophan.

Protein modifications produced by ROS, RNS and RXS can be classified as transient, reversible or irreversible. Reactions of free radicals with proteins leads to formation of protein radicals, which are generally short-lived, transient and are not useful as biomarkers. Protein hydroperoxides formed upon reactions with ROS are also unstable and decompose forming more stable products [9,10]. Examples of reversible modifications are cysteine (Cys) thiol oxidation to sulfenic acid, methionine (Met) oxidation to methionine sulfoxide or cysteine *S*-nitrosylation and *S*-glutathionylation (Table 1, Fig. 1). While these modifications are of vital importance for regulation of protein function and metabolic processes, they are of less importance as permanent markers of OS/NS/XS, so this review will concentrate on irreversible protein modifications.

**Table 1:** Most important oxidative, nitrative and chlorinative modifications of proteins. After [11] modified.

Amino acid	Modification	Stability/Reversibility
Cysteine	Oxidation of –SH to sulfenic acid (-SOH), sulfinic acid (-SO_2_H) or sulfonic acid (-SO_3_H) Formation of a disulfide bond –SS-	First stage, and in some cases second stage reversible Reversible
Cysteine	Nitrosylation [formation of (-SNO)]	Reversible
Cysteine	Glutathionylation	Reversible
Tyrosine, tryptophan, other amino acids	Protein radicals	May be reduced or react to form further products
Glutamic acid, tyrosine, lysine, leucine, valine, proline, isoleucine	Hydroperoxides	May be reduced; decompose to further products
Histidine	2-Oxohistidine	Irreversible
Lysine, arginine, proline, threonine	Formation of carbonyl derivatives by direct oxidative attack on amino-acid side chains (α-aminoadipic semialdehyde from lysine, glutamic semialdehyde from arginine, 2-pyrrolidone from proline, and 2-amino-3- ketobutyric acid from threonine)	Decarbonylation [?]
Lysine, cysteine, histidine	Formation of carbonyl derivatives by secondary reaction with reactive carbonyl compounds derived from oxidation of carbohydrates (glycoxidation products), lipids (MDA, 4-HNE, ACR) and advanced glycoxidation and lipoxidation end products	Irreversible
Methionine	Methionine sulfoxide	Reversible by methionine sulfoxide reductases
Phenylalanine	*o*-Tyrosine, *m*-tyrosine	Irreversible
Tyrosine	Hydroxylation to 3,4-dihydroxyphenylalanine Dimerization to dityrosine	Irreversible
Tyrosine, tryptophan, histidine	Nitration [introduction of (-NO_2_)]	Irreversible [Denitration ?]
Tyrosine	Chlorination to 3-chlorotyrosine	Irreversible
Tryptophan	5-Hydroxytryptophan, 7-hydroxytryptophan, kynurenine, N-formylkynurenine	Irreversible

## FORMATION OF NON-ENZYMATICALLY MODIFIED PROTEINS

Compared to other oxidative modifications, carbonyls are relatively difficult to induce and in contrast to, for example, methionine sulfoxide and cysteine disulfide bond formation, carbonylation is an irreversible oxidative process [11]. Protein carbonylation is an oxidative modification induced by ROS, RNS, RXS and reactive aldehydes. It consists in formation of reactive aldehyde or ketone residues on proteins, which can react with 2,4-dinitrophenylhydrazine (DNPH) forming hydrazones. There are two ways of protein carbonylation. "Primary protein carbonylation" is due to oxidation of some amino acid residues, initiated by ROS, RNS and RXS, often catalyzed by metals while “secondary protein carbonylation” is caused by addition of aldehydes. The aldehydes are formed mainly in the process of lipid peroxidation [malondialdehyde, MDA; 4-hydroxy-2,3-*trans*-nonenal, (4-HNE); 2-propenal (acrolein, ACR)], but may be also by-products of glycolysis and the glycation process (methylglyoxal, glyoxal).

In the first pathway, ROS, RNS and RXS directly attack the protein producing, eventually, highly reactive carbonyl derivatives by oxidation of the side chains of lysine (Lys), arginine (Arg), proline (Pro), and threonine (Thr) residues, particularly *via* metal-catalysed oxidation, from the cleavage of peptide bonds in the α-amidation pathway or by oxidation of glutamyl residues. The main carbonyl products of metal-catalysed protein oxidation are glutamic semialdehyde, a product of oxidation of Arg, aminoadipic semialdehyde, a product of Lys oxidation, 2-pyrrolidine, a product of histidine (His) oxidation and 2-amino-3-ketobutyric acid, a product of oxidation of Thr (Fig. 1E) [12]. Carbonylation is site-specific; an iterative statistical method has been proposed to identify potential sites of carbonylation [13].

The second type of reaction involves the addition of reactive aldehyde groups to the side chains of Cys, His, or Lys residues via Michael addition (Fig. 1F). Reactive carbonyl groups can be also generated through the reaction of the amino group of lysine residues with reducing sugars or their oxidation products (glycation/glycoxidation products) [14].

Dimerization of tyrosyl radicals (Tyr^•^) leads to the formation of dityrosine (Fig. 1H). Products of oxidative destruction of tryptophan (Try) include kynurenine and N-formylkynurenine (Fig. 1I). All these products have their characteristic fluorescence and their content can be easily evaluated fluorimetrically [15,16].

RNS can oxidize proteins and alter their biological functions also in other ways. Nitration of amino acids, such as tyrosine (Tyr) and, to a lesser extent, Try and His, is an important form of protein modification that occurs during NS [17]. Tyr, a nonessential aromatic amino acid, carrying a hydroxyl group, is often exposed at the surface of proteins, making them vulnerable to nitration, as well as oxidation [18,19].

The nitration of Tyr is mediated by RNS such as ONOO^-^/ONOOH and ^•^NO_2_ although nitration can also by accomplished by heme peroxidases and nitrite [20]. The two main mechanisms of biological nitration, the ONOO^-^/ONOOH and the heme peroxidase pathways, lead both to the formation of Tyr^•^ and ^•^NO_2_, which combine with diffusion controlled rates to form 3-nitrotyrosine (3-NT; Fig. 1H). The oxidants leading to Tyr^•^ formation include CO_3_^•-^, ^•^OH or oxo–metal complexes. Importantly, ^•^NO_2_ alone is inefficient in promoting nitration, because its reaction with Tyr to produce Tyr^•^ is slow compared to other processes that ^•^NO_2_ undergoes. I. a., reaction with another Tyr^•^ to form 3,3-dityrosine competes with the formation of 3-NT. However, under certain conditions protein radicals can be stabilized, e. g. when intra- and intermolecular dimerization is limited due to diffusional and spatial constraints, both in aqueous and hydrophobic compartments. In such cases reaction of Tyr^•^ with ^•^NO_2_ may be favoured. Another pathway competing with Tyr nitration is the formation of 3-hydroxytyrosine, which can be performed mainly by ^•^OH or oxo–metal complexes. An alternative radical mechanism for Tyr nitration involves the reaction of a Tyr^•^ with ^•^NO to form 3-nitrosotyrosine followed by two-electron oxidation to 3-NT [21].

Hypochlorous acid (HOCl) is the main player involved in protein chlorination *in vivo* [16]. HOCl is generated by the reaction of H_2_O_2_ with chloride ions (Cl^-^) catalysed by myeloperoxidase (MPO, EC 1.11.1.7) [22–24]. For a long time, myeloperoxidase (MPO) was regarded as the only human enzyme known to produce HOCl at the physiological concentrations of chloride (100-140 mM) [25]. Nevertheless, recent findings revealed that another mammalian heme peroxidase, peroxidasin 1, is capable of catalysing the oxidation of chloride to HOCl, too. The enzyme is also known as vascular peroxidase 1 [26–29]. Up to 80% of the H_2_O_2_ generated by activated neutrophils may be used to produce local concentrations as high as 20-400 µM HOCl within an hour [30,31]. The pK*_a_* of HOCl is 7.59 [32], so at physiological pH values, HOCl exists in equilibrium with its anion ^-^OCl at approximately equal concentrations. HOCl is a powerful oxidant and plays an important physiological role. MPO-produced HOCl is involved in innate immune response and kills invading pathogens [33,34]. Green et al. [35] showed that the diminution of HOCl production observed with decreasing Cl^-^ availability results in impaired killing of bacteria. However, during chronic inflammation the excessive production of HOCl leads to the host tissue damage and plays a pathophysiological role in inflammatory diseases [36]. Proteins are major targets for HOCl, and the reactions of this oxidant with proteins result in side-chain modifications (mainly chlorination of Tyr residues, Fig. 1H), cross-linking and backbone fragmentation [37,38].

## PROTEIN CARBONYLATION IN AGING AND AGE-RELATED DISEASES

Protein carbonyl content is the most general and broadly used biomarker of oxidative protein damage and, more generally, OS. However, protein carbonyls are important not only as a biomarker for protein oxidation in aging and disease. They have also been shown to impair protein structure and function and to participate in the etiology and progress of diseases and age-related changes in the body [39,40]. Carbonylation may alter the conformation of the polypeptide chain, which leads to partial or total inactivation of proteins. The consequent loss of function or structural integrity of carbonylated proteins can have a wide range of downstream functional consequences and may underlie the subsequent cellular dysfunctions and tissue damage [41]. Protein carbonylation was demonstrated to modify activities of enzymes and other protein functions like DNA binding of transcription factors [42]. Carbonylation can lead to functional impairment of proteins involved in insulin signaling, so the insulin signaling pathway gets disrupted by carbonylation [43].

Another mechanism of protein carbonyl action involves inhibition of proteasomal activity. While moderately carbonylated proteins are degraded by the proteasomal system, heavily carbonylated proteins form high-molecular-weight aggregates that are not digested and accumulate. Such aggregates of carbonylated proteins are resistant to degradation and can inhibit proteasomes. Neurodegenerative diseases are directly associated with the accumulation of proteolysis-resistant aggregates of carbonylated proteins in tissues [39].

It has been hypothesized that protein carbonylation is reversible, and protein carbonyls can be removed by a "decarbonylase" activity; thus, protein carbonylation can play a role in cellular signaling. Thioredoxin was postulated to be involved in protein decarbonylation [44–46]. However, experimental support for this hypothesis is scarce. Age-related increase in the protein carbonyl content has been demonstrated in many objects. Data from various laboratories demonstrated a dramatic increase in the content of carbonylated proteins during the last third of the lifespan of various objects, i. a., human dermal fibroblasts in culture [47], human lens [48], rat liver [49], house fly [50] and *Caenorhabditis elegans* [51]. Further examples of age-related increase in the level of protein carbonyls are given in Table 2.

**Table 2:** Examples of studies on the effect of aging on protein carbonyl level.

Problem studied	Material or object studied/methods	Findings	Reference
Effect of replicative aging of fibroblasts *in vitro* on protein carbonyl level	WI-38 fibroblasts, intermediate or middle-aged (PD between 25 and 39) and replicatively senescent (PD < 40)/OxyBlot	Increase in the level of carbonylated proteins, Preferential carbonylation of certain proteins	[52]
Effect of replicative aging and heat stress on protein carbonyl level in human fibroblasts	Human foreskin fibroblasts, middle-aged and senescent/ OxyBlot	Increased protein carbonyl content in senescent cells and in heat stressed cells, without recovery	[53]
Effect of aging and late onset dietary restriction on the protein carbonyl level in cerebral hemispheres	BALB/c mice, 4-w old and 84-w old/DNPH assay and WB	Increased protein carbonyl level in old mice, Reduction of protein carbonyl level after 3-m calorie restriction	[54]
Effect of aging on protein carbonyl level of high-molecular weight protein aggregates isolated from the bone marrow and splenic cells	Female C57BL/6 J mice/Oxyblot, Protein Carbonyl Assay kit	Enhanced protein carbonyl level in 22-m old vs 3-m and 12-m old mice	[55]
Effect of aging on protein carbonyl level of testis mitochondria	5-m vs 30-m old rats/2D PAGE, WB, carbonyl detection with biotin-hydrazide	Age-related increase in the carbonyl content of many proteins, decrease for some proteins	[56]
Comparison of protein carbonyl content in mitochondria of slow-twitch and fast-twitch muscles	Fisher 344 female rats/MS	Fast-twitch muscle contain twice as many carbonylated mitochondrial proteins as slow-twitch muscle	[57]
Effect of age on protein carbonyl content of erythrocyte membranes	49 healthy subjects of both sexes aged 17- 80 y/DNPH assay	High correlation between age and protein carbonyl content, Negative correlation between protein carbonyl content and total antioxidant capacity of plasma (FRAP)	[58]
Effect of age on carbonyl content of mouse liver	Young (3 m) and aged (24 m) male C57BL/6 mice/2D PAGE, WB	Increased protein carbonylation in aged mice, especially of BiP/Grp78, protein disulfide isomerase (PDI) and calreticulin	[59]
Effect of age on protein carbonyl level in rat cerebral cortex and hippocampus	4‑m, 12‑m and 22‑m old rats/DNPH assay	Higher protein carbonylation in hippocampus than in cerebral cortex, Increase in protein carbonyl level with age, attenuated by physical exercise	[60]
Effect of age and sarcopenia on carbonyl content of skeletal muscle subfractions	Sarcoplasmic, myofibrillar, and mitochondrial subfractions from *musculus* *vastus lateralis* biopsies of 16 young and 16 elderly persons/ Oxyblot	Increased mitochondrial (but not myofibrillar or sarcoplasmic) protein carbonyl content with aging, No effect of stage I sarcopenia	[61]
Effect of age and gender on protein carbonyl content in saliva and plasma	273 healthy Chinese subjects, aged between 20 and 79/ELISA	Significant correlation of saliva and plasma protein carbonyls with age, No relation to gender	[62]
Effect of aging on protein carbonyl level of mouse skeletal muscles	Muscles from 3, 15, 24, 27 and 29 m old female C57Bl/6J mice/DNPH assay	Protein carbonyl level of gastrocnemius muscles unchanged between 3 and 15 m, increasing at 27 and 29 m. No age-related decrease in protein thiol level or increase in the levels of MDA and F2-isoprostanes	[63]
Effect of age and physical exercise on the carbonyl level of plasma proteins	481 participants of both sexes aged 65-69 y and 239 participants aged 90 y or more/ELISA	Elevation of protein carbonylation with aging, attenuated by physical activity	[64]
Effect of aging on the level of protein carbonyls in human rectus abdominis and vastus lateralis muscles	Muscle biopsies of 11 children 0-12 y old and 11 persons 52-76 y old/2D PAGE, WB	No significant differences in the global level of protein carbonyls between the groups in both rectus abdominis and vastus lateralis muscles	[65]
Effect of age on protein carbonyl content of external intercostals and quadriceps muscles	12 young and 12 elderly persons of both sexes/ DNPH assay	Increased levels of protein carbonyls in external intercostals of elderly women, but not of elderly men	[66]

DNPH, dinitrophenylhydrazine; 2D PAGE, two-dimensional polyacrylamide gel electrophoresis; MS, mass spectrometry, PD, population doublings; WB, Western Blotting

Many studies have demonstrated the existence of a relationship between the level of protein carbonylation in cells or tissues/organs and human or animal age and lifespan, as exemplified below. The level of protein carbonylation in human fibroblasts have been found to increase exponentially with advancing age of donors [67]. When mobility of fruit flies *Drosophila melanogaster* was restricted by culturing under conditions preventing flying, the lifespan of the insects was increased 2 to 3-fold. Age-related accumulation of protein carbonyls was slower in mobility-restricted flies in comparison with control flies, which could fly and had shorter lifespan [50]. Transgenic fruit flies which overexpressed CuZn-superoxide dismutase (SOD1) and catalase had prolonged lifespan. Accumulation rate of protein carbonyls in these flies was slower than in control flies whose lifespan was shorter [68]. Calorie restriction was also demonstrated to slow down the rate of protein carbonyl accrual. In calorie-restricted mice which had lifespan increased by 35%, the rate of accumulation of protein carbonyls in several tissues was decelerated [69,70].

Protein carbonylation is selective with respect to the protein and this rule refers also to carbonylation during aging and in age-related diseases. Approximately 10% of the proteome is more prone to carbonylation during ageing or disease than other proteins [71,72]. In the brains of patients affected with Alzheimer's disease (AD) and Parkinson’s disease (PD), mitochondrial MnSOD superoxide dismutase (SOD2) is one of the major targets of oxidative damage [73]. In turn, aconitase was found to be the only protein in the mitochondrial matrix that exhibited an age-associated increase in carbonylation in *D. melanogaster*. The accumulation of carbonyl groups was accompanied by an approximately 50% loss in aconitase activity [74]. Interestingly, the set of proteins that become carbonylated differs in various species. For example, aging-associated protein carbonylation was only seen in two proteins in mouse blood plasma, albumin and transferrin, while in the rat plasma, only albumin and α-macroglobulin showed significant progressive age-dependent carbonylation [75]. There are several possible explanations for this specificity of carbonylation. One is the presence of a transition metal on the protein, another being the localisation of proteins to be carbonylated close to ROS generating sites. However, in general the molecular basis for the apparent specificity of protein carbonylation still remains unclear [76].

Elevation of the protein carbonyl level has been reported for many diseases. Protein carbonylated levels are widely used index to determine the extent of oxidative modification of proteins both under *in vivo* and *in vitro* conditions. Increased protein carbonyl levels were found in the cerebrospinal fluid of patients with multiple sclerosis [77] and in blood plasma of patients with multiple sclerosis [16] as well as myasthenia gravis [78]. In multiple sclerosis, increased level of protein carbonyls was also found in the brain white and gray matter [79].

The large volume of literature and heterogeneity of results makes a comprehensive understanding of the changes occurring in human brain in AD elusive. In AD, the increase in protein carbonylation level was different in various regions of the brain. It was increased by 42% in hippocampus and by 37% in the inferior parietal lobule, with respect to cerebellum, which shows little degenerative changes in this disease [80]. Some specifically carbonylated proteins in AD brain were identified in different stages of the process, including the exacerbate mild cognitive impairment (MCI) and early AD stages [81–84].

What’s more, a new meta-analysis deﬁnes the pattern of changes in OS related markers by brain region in human AD and MCI brain tissue. Protein carbonylation was signiﬁcantly increased in the occiput and in the hippocampus in AD, while there were no signiﬁcant changes noted in other brain regions [85]. Shen et al. evaluated the levels of total protein carbonyls and identified the oxidative modification proteins in the sera of 3×transgenic AD mice. Their results suggested that OS is an early event in the development of AD, and analysis of specific serum protein oxidation may be more plausible for the search of AD biomarkers [86]. Brain samples of patients with Huntington disease showed increased carbonylation of more than a dozen of proteins including glial fibrillary acidic protein, aconitase, enolase 1 and creatine kinase B, glycolytic enzymes and mitochondrial proteins related to ATP production [87]. Increased level of protein carbonyls was also found in the spinal cord of G93A-SOD1 transgenic mice, an animal model of amyotrophic lateral sclerosis (ALS) [88].

Numerous data point to the role of RCS as both propagators and products of oxidative damage in neurodegenerative diseases, especially in AD [89]. In AD, the concentration of free 4-HNE was reported to be increased in the plasma and cerebrospinal fluid [90], whereas ACR content was higher in the amygdala and hippocampus/parahippocampal gyrus of AD patients [91]. MDA accumulation has been detected in the cytoplasm of astrocytes and neurons in both normal ageing and in AD patients [92]. Increased concentrations of MDA (in blood plasma and serum) and 4-HNE (in the plasma and cerebrospinal fluid) have also been reported for PD patients [91]. 4-HNE levels are significantly elevated in the sera and spinal fluid of ALS patients and positively correlate with the extent of the disease but not with the rate of progression, which suggest 4-HNE and carbonyl bearing 4-HNE-protein adducts as possible biomarkers of the disease [77,93].

### Methods of protein carbonylation analysis

The generally used method of quantifying carbonyl groups is based on the reaction with 2,4-dinitrophenylhydrazine (DNPH). This compound reacts with carbonyl groups, forming the stable 2,4-dinitrophenylhydrazone. Dinitrophenyl group (DNP) adduct can be detected by different methods. The DNP group itself absorbs ultraviolet light, so the total carbonyl content of a protein or mixture of proteins can be quantified by a spectrophotometric assay [94]. Alkalinization of the medium may bring absorption maximum of the DNP group from 370 nm (UV) to 450 nm (visible region) [95].

The assay of carbonylated proteins has been simplified by the availability of commercial antibodies specific for DNP, which allow for their detection by immunoblotting. Dot blot analysis allows for a very sensitive quantification of the total level of protein carbonylation in a sample [96]. Immunoblotting assays based on the use of anti-DNP antibodies have been developed as an attempt to identify oxidatively damaged proteins in human tissues and body fluids. The carbonyl content in individual proteins is estimated by one-dimensional (1D) or two-dimensional (2D) sodium dodecyl sulfate (SDS) gel electrophoresis followed by Western blot immunoassay (Oxyblot). These two methods have significantly higher sensitivity and specificity than all other total carbonyl assays, but are still semiquantitative [11]. An alternative to immunochemical detection of DNP derivatives of carbonylated proteins is the reaction with a fluorescent reagents reacting with carbonyl groups such as fluorescein-5-thiosemicarbazide [97] or fluorescent hydrazides [98,99]. Fluorescent hydrazides such as coumarin hydrazine were also used for detection of protein carbonyls in living cells [100].

DNP assay of protein carbonyls can be also combined with protein fractionation by high-performance liquid chromatography (HPLC) to obtain better sensitivity and specificity than measuring total carbonyls in a protein mixture [11]. Mass spectrometry (MS) allows for precise identification of carbonylated proteins and characterization of the carbonylation sites [101]. Proteomic tools provide a promising way to decode disease mechanisms at the protein level and help to understand how carbonylation affects protein structure and function. Recently, Havelund et al. (2017) proposed a peptide-centric approach for identification and characterization of up to 14 different types of carbonylated amino acids in proteins. The use of diagnostic biotin fragment allows MS/MS data analysis to pinpoint sites of biotin labeling and improve the confidence of carbonyl peptide assignments [102].

## NITRATIVE PROTEIN MODIFICATIONS IN AGING AND AGE-RELATED DISEASES

It should be noted that protein Tyr nitration is observed *in vivo* in healthy tissues, indicating that there is a basal flux of RNS; nevertheless, physiological nitration levels are typically low. Possible biochemical consequences of protein Tyr nitration involve changes in activity (usually loss, but sometimes gain of function), induction of immune responses, interference with tyrosine-kinase-dependent pathways, alteration of protein assembly and polymerization, and effects of protein turnover: either facilitation of protein degradation or induction of formation of proteasome-resistant protein aggregates, depending on the dose [103,104].

Furthermore, protein Tyr nitration is also associated with physiological aging and pathophysiology of several age-related diseases such as atherosclerosis, multiple sclerosis, AD, PD, ALS, cystic fibrosis, asthma, lung diseases, myocardial malfunction, stroke, chronic hepatitis, cirrhosis, diabetes, etc [105]. Increased content of nitrates, nitrites, and free 3-NT were also found in the cerebrospinal fluid of subjects with neurodegenerative diseases and have been proposed as functional biomarkers of neurodegeneration [16,106].

### Two faces of ^•^NO: implications for brain aging

Nitric oxide and other RNS appear to play crucial roles in the brain such as neuromodulation, neurotransmission and synaptic plasticity, but are also involved in pathological processes such as neurodegeneration and neuroinflammation. Nitric oxide is a short-lived gaseous physiological messenger, which is highly diffusible and lipophilic in nature [107]. As an important neurotransmitter and signaling molecule, ^•^NO is involved in numerous physiological processes throughout the nervous system. Apart from guanylate cyclase, ^•^NO targets include ion channels, which are involved in setting neuronal excitability and calcium homeostasis, additional to its involvement in physiological plasticity processes (long-term potentiation; long-term depression), which can include the N-methyl-D-aspartate receptor-mediated calcium-dependent activation of neuronal ^•^NO synthase [108].

Nevertheless, ^•^NO possesses a controversial effect on cell viability by acting both in protection against apoptogenic stimuli, and by inducing apoptosis when produced at elevated concentrations. Moreover, excessive generation of ^•^NO, favors the formation of reactive ONOO^-^/ONOOH and NO_2_ species that can mediate nitration of aging brain proteins [109]. Acute and chronic inflammation result in increased ^•^NO formation and NS. It is well documented that ^•^NO and its toxic metabolite, ONOO^-^/ONOOH, can inhibit components of the mitochondrial respiratory chain leading to cellular energy deficiency and, eventually, to cell death. Within the brain, the susceptibility of different brain cell types to ^•^NO and ONOO^-^/ONOOH exposure may be dependent on factors such as the intracellular glutathione (GSH) concentration and cellular stress resistance signal pathways [110].

### ^•^NO toxicity: regulation by glutathione

GSH is the most abundant low molecular weight thiol in mammalian cells and acts as the major cellular antioxidant. Aquilano et al. [111] reported that GSH may constitute the most important buffer of ^•^NO toxicity in neuronal cells, and demonstrated that the disruption of cellular redox buffering controlled by GSH makes neuronal cells susceptible to endogenous physiological flux of ^•^NO. GSH levels in the brain decline progressively during aging and in neurodegenerative disorders, such as AD or PD [112,113]. It has been proposed that the decrease in GSH concentration could be mainly a consequence of the formation of protein mixed disulfides. The intracellular depletion of GSH can induce cellular stress in ^•^NO-producing cells through a ^•^NO-dependent mechanism, resulting in such effects as induction of DNA damage, inhibition of cytochrome *c* oxidase activity, accumulation of S-nitrosocysteine and increased nitration of protein Tyr residues. What’s more, ^•^NO seems to be the main mediator of cell proliferation arrest through the extracellular signal-regulated kinase-1/2-p53 signaling pathway and apoptosis through the translocation of mitochondrial apoptosis-inducting nuclear factor [111].

### Tyrosine nitration of synaptic proteins

Synaptic proteins can undergo extensive posttranslational modifications. Numerous evidence suggests that aging and diseases can induce nitrative stress via excessive ^•^NO production. NS can lead to uncontrolled *S*-nitrosylation/Tyr nitration, which can represent crucial pathological features that contribute to the onset and progression of various neurodegenerative diseases, including AD or PD [110,111].

It has been suggested that phosphorylation and nitration of protein Tyr residues plays a role in signaling pathways at the nerve terminal and affects functional properties of proteins involved in the synaptic vesicle (SV) exo-endocytotic cycle [114]. Protein conformational changes induced by ^•^NO have strong impacts on protein-protein interactions in the docking/fusion steps of vesicle release. Depending on the concentration of ^•^NO and the reversibility of protein nitration, the consequences for neuronal signaling are important and relevant in physiology and pathology. According to Di Stasi and coworkers [115], ONOO^-^/ONOOH causes Tyr nitration of SNAP-25 and Munc-18, two presynaptic proteins, which are involved in sequential steps leading to vesicle exocytosis. Notably, these effect were strongly reduced in the presence of NaHCO_3_, indicating that ONOO^-^/ONOOH acts mainly intracellularly. Synaptophysin, one of the most abundant integral proteins of SV membrane, can be also nitrated (on Tyr250) and the formation of the synaptophysin/dynamin complex is impaired following ONOO^-^/ONOOH exposure [114,116]. Mallozzi et al. [114] have identified by LC–MS/MS analysis one major nitration site at Tyr354 in dynamin I isolated from synaptosomes treated with ONOO^-^/ONOOH. LC–MS/MS analysis revealed also that in untreated synaptosomes dynamin I showed a basal level of nitration on Tyr125, Tyr541 and Tyr669; however, the low physiologic nitration level of these sites did not affect dynamin I functional properties. Instead, Tyr354 was nitrated only after ONOO^-^/ONOOH treatment of synaptosomes implying that this site-specific post-translational modification likely accounts for dynamin I dysfunction. Vrljic et al. [117] detected nitration in 6 of 11 surface accessible Tyr residues of synaptotagmin 1 [three in the C2A domain (Tyr151, Tyr216 and Tyr229) and three in the C2B domain (Tyr311, Tyr364 and Tyr380)]. Synaptotagmin 1 is a Ca^2+^ sensor for SNARE (soluble N-ethylmaleimide sensitive factor attachment protein receptor)-mediated, Ca^2+^-triggered synaptic vesicle fusion in neurons. Integration of the peak intensity for the individual synaptotagmin 1 peptides suggests a stoichiometry for the 3-NT modifications of 1–10% depending on the site, with the exception of Tyr151, which appears to be ∼100% modified since an unmodified form was not identified [108].

Amyloid beta (Aβ) is a critical factor involved in the pathogenesis of AD. It was demonstrated that continuous intracerebroventricular infusion of Aβ1–40 induced a time-dependent expression of the inducible nitric oxide synthase (iNOS) and an overproduction of ^•^NO in the rat hippocampus. The pathophysiological significance of the overproduction of ^•^NO for brain function was manifested by an impairment of nicotine-evoked acetylcholine (ACh) release and memory deficits [118]. Tran et al. [119] found that chronic Aβ 1–40 infusion caused a robust ONOO^-^/ONOOH formation and subsequent Tyr nitration of hippocampal proteins. Immunoprecipitation and Western blot analyses revealed that synaptophysin is a main target of Tyr nitration. These findings suggest that the Tyr nitration of synaptophysin is related to Aβ-induced impairment of ACh release.

### Tyrosine nitration of brain proteins

Nitric oxide participates in the regulation of the daily activities of cells as well as in cytotoxic events. Tyr nitration is one specific form of protein modification that is associated with age-related neurodegenerative diseases [120]. Protein nitration enhances Aβ aggregation in a rodent model of AD [109]. By mediating Tyr nitration at the *ortho* position, ONOO^-^/ONOOH modification of proteins can block later phosphorylation events, thereby inducing protein dysfunction [121], some researchers have proposed that dynamic interplay between nitration and phosphorylation may be required for some normal biological functions, and Tyr nitration can contribute to differentiation of neuronal cell types and to neurite elongation.

The microtubule-associated tau protein is unfolded and finely soluble under physiological conditions, but in the brain tissue of AD changes in its conformation occur, affecting its solubility. Horiguchi et al*.* demonstrated the presence of nitrated tau protein in pretangles, neurofibrillary tangles, as well as tau inclusions in AD brain. Tau contains five Tyr residues (located at 18, 29, 197, 310, and 394), that can undergo nitration to initiate a range of ‘tauopathies’ [122]. In AD patients, the N-terminal tyrosine residues of the tau protein (Tyr18 and Tyr29) are more susceptible to nitrative modifications than other tyrosine residues (Tyr197 and Tyr394) [123]. Tau nitration at Tyr197 and Tyr18 has been reported to enhance disease progression in a range of neurodegenerative disorders [124], whereas nitration at Tyr29 appears to be a specific characteristic of AD [123].

It was found that nitration of other proteins perturbs pH regulation, energy metabolism, and mitochondrial functions, and may be involved in the mechanisms of neuronal loss and progression of AD. In particular, such nitrated proteins were identified in the AD hippocampus as α-enolase, carbonic anhydrase II, glyceraldehyde-3-phosphate dehydrogenase, ATP synthase α-chain and voltage dependent anion channel protein 1 (VDAC-1), using a redox proteomics approach. Nitration of ATP synthase α-chain and VDAC-1 is associated with mitochondrial dysfunction and neuronal cell death in the AD hippocampus. Moreover, nitrated proteins are usually tagged for selective destruction in proteasomes, but in AD this pathway may be defective due to oxidation of ubiquitin carboxy-terminal hydrolase L-1 in the inferior parietal lobule and hippocampus [122,125,126]. Main brain proteins nitrated in AD are listed in Table 3.

**Table 3:** Selected proteins nitrated in Alzheimer’s disease.

Nitrated protein	Material	Methodology	Major observations	Ref.
Enolase	Male Wistar rat synaptosomes	WB	Nitration of enolase and synaptic proteins mediated by H_2_O_2_, ^*^NO_2_ and amyloid β heme peroxidase activity	[127]
Nitro-triosephosphate isomerase (nitration of tyrosines 164 and 208, close to the catalytic site)	Immunoprecipitates from hippocampus (9 individuals) and frontal cortex (13 individuals) of AD patients, compared with healthy subjects (4 and 9 individuals, respectively); Human embryonic kidney cells overexpressing mutant triosephosphate isomerase	WB, Transmission electron microscopy, Atomic force microscopy	Nitro-triosephosphate isomerase forms large beta-sheet aggregates *in vitro* and *in vivo*, Nitro-triosephosphate isomerase binds tau monomers and induces tau aggregation to form paired helical filaments	[128]
Brain proteins	Brain samples; normal control subjects: 4 females and 2 males, average age at death of 81 ± 6.4 y; amnestic mild cognitive impairment (MCI) patients, 4 females and 2 males, average age at death of 88 ± 3.8 y	Slot blot, Immunohistochemistry	Protein nitration is higher in the inferior parietal lobule (IPL) and hippocampus in MCI than in control subjects	[120]
α-Enolase, triosephosphate isomerase, neuropolypeptide h3, β-actin, L-lactate dehydrogenase, ɣ-enolase	IPL tissue specimens used for analyses taken at autopsy from five AD patients and five control subjects	WB, MS	Identification of six targets of protein nitration in AD suggests a role of protein modification by RNS in the progression of AD	[129]
α-Enolase, glyceraldehyde-3-phosphate dehydrogenase, ATP synthase alpha chain, carbonic anhydrase-II, voltage-dependent anion channel-protein (hippocampus)	Hippocampal samples from six AD patients and six age-matched controls	Immunoprecipitation, WB, MS	Nitration of proteins in AD hippocampus may be involved in the mechanisms of AD	[130]
Peroxiredoxin 2, triose phosphate isomerase, glutamate dehydrogenase, neuropolypeptide h3, phosphoglycerate mutase 1, H^+^– transporting ATPase, α‐enolase, fructose‐1,6‐bisphosphate aldolase	IPL samples from four early AD (EAD) patients (79 ± 2 years) and four age‐matched controls (average age at death of 86 ± 4 years).	WB, 2D PAGE, In‐gel trypsin digestion, MS	The level of nitrated proteins in the IPL of early AD patients increased by 18% increase compared with age-matched controls	[131]

2D PAGE, two dimensional polyacrylamide gel electrophoresis; IPL, inferior parietal lobule;MS, mass spectrometry; WB, Western Blotting

It should be noted that aging is an important risk factor for human α-synucleinopathies such as PD. There is a link between aging, α-synuclein (αSyn) abnormalities and enhanced vulnerability to neurodegenerative processes. It was also reported that phospho-Ser 129 and nitrated αSyn are formed within dopaminergic neurons of the monkey *substantia nigra* in the course of normal aging [132]. Schildknecht et al. [133] hypothesized that under physiological conditions αSyn may act as an intracellular scavenger of oxidants, catalytically regenerated, and performs an important protective role before the onset of disease or during aging. αSyn is a 140 amino-acid protein, originally identified in association with synaptic vesicles in the presynaptic nerve terminals and has been shown to interact with membranes both *in vitro* and *in vivo*. It is predominantly expressed in the brain (in the neocortex, hippocampus, *substantia nigra*, thalamus, and cerebellum, accounting for approximately 1% of brain weight) and is also present in other cells and tissues, including erythrocytes [134]. αSyn is involved in the modulation of synaptic activity through regulation of assembly of SNARE-complex of presynaptic vesicles, regulation of neurotransmitter release, , regulation of cell differentiation and phospholipid metabolism [135]. Susceptibility to PD may be linked to modulation of αSyn protein expression. Furthermore, nitration of αSyn was associated with enhanced propensity of this protein to aggregate. Burai et al. [136] examined the site-specific incorporation of 3-NT at different regions of αSyn. They found that depending on the site of nitration, various nitrated αSyn species exhibit distinct structural and aggregation properties and exhibit reduced affinity to negatively charged vesicle membranes. Intermolecular interactions between the N- and C-terminal regions of αSyn play critical roles in mediating nitration-induced oligomerization of αSyn. In mutants, in which Tyr39 is not available for nitration, the extent of cross-linking is limited mostly to dimer formation, whereas mutants in which Tyr39 is available, along with one or multiple C-terminal tyrosines remain nitrated. Nitrated αSyn was observed to induce adaptive immune responses that exacerbate PD pathology in the mouse MPTP model of PD [137]. Increased nitrated αSyn is present in peripheral blood mononuclear cells of idiopathic PD patients compared to healthy individuals [138]. These studies provide evidence for a direct link between nitrative damage and the onset and progression of neurodegenerative synucleinopathies. More recently, Kleinknecht et al. [139] reported that αSyn can be nitrated and form stable covalent dimers originating from covalent crosslinking of two Tyr residues. Nitrated Tyr residues, but not dityrosine-crosslinked dimers, contribute to αSyn cytotoxicity and aggregation. Analysis of Tyr residues involved in nitration and crosslinking revealed that the C-terminus, rather than the N-terminus of αSyn, is modified by nitration and dityrosine formation. These data suggest that C-terminal Tyr133 plays a major role in αSyn aggregate clearance by supporting the protective Ser129 phosphorylation for autophagy and by promoting proteasomal clearance. C-terminal Tyr nitration increases pathogenicity and can only be partially detoxified by αSyn dityrosine dimers. It seems that complex interplay between Ser129 phosphorylation and C-terminal Tyr modifications of αSyn likely participates in PD pathology. Table 4 shows data on α-synuclein nitration in PD.

**Table 4:** α-Synuclein nitration in Parkinson’s disease.

Nitrated Protein	Materials	Methodology	Major observations	Reference
αSyn	Male Fischer 344 rats, 3-month-old vs 16-month-old	Western blotting, ELISA	Microglia activation and proinflammatory cytokine expression enhanced in the *substantia nigra* of elderly rats following intrapallidal lipopolysaccharide administration, Greater nitration of αSyn in the *substantia nigra* of 16-month-old rats vs 3-month-old rats, accompanied by a higher expression level of iNOS	[140]
αSyn	Twelve-month-old male nTg, SYN Tg, and SYN-null mice; primary neuronal and glial cultures.	WB, Immunostaining, Sequential biochemical fractionation, Immunoelectron microscopy	Neuroinflammation and Syn pathology are linked mechanistically to the onset and progression of PD	[141]
α-Syn (nitration of Tyr125 and Tyr136)	Squirrel monkeys of 2 age groups: <10 y (6–9 years, n=4) and >16 (17–19 y, n=3).	Immunohistochemistry	Age-related elevations of modified protein	[132]

Tg, transgene; WB, Western blotting

### Nitration and aging

It has been extensively documented that increased nitration is often connected to the development of age-related diseases. High concentrations of peroxynitritrous acid may affect modulation of mitochondrial respiration that can act as platform for development of prevalent neurodegenerative diseases. Proteomic analysis by ESI-MS/MS had shown that flotillin-1 and α-tubulin are nitrated in the rat in the course of aging. Age dependent accumulation of 3-NT on skeletal muscle glycogen phosphorylase b (Ph-b) is reported in an experimental rat model (106, 140). Results of selected studies on protein nitration in aging are shown in Table 5.

**Table 5:** Selected results of nitroproteomic studies of aging.

Material	Methods	Nitrated Protein	Findings	Reference
Male Wistar rats aged 6 m (adult) and 25 m (old)	WB	Quadriceps protein fractions	3-NT levels higher in all protein fractions of skeletal muscle in old male rats, especially in the mitochondrial fraction	[165]
Young (19–22 w) and old (24 m) C57BL//6 male mice	SDS PAGE, WB	Hepatic proteins	Significantly higher level of Tyr nitration of proteins in old mice vs young mice	[166]
PC12 cell culture	WB, MS	Actin, tubulin, Hsp70, Hsp90	ONOOH-treated Hsp70, actin, and tubulin nontoxic for motor neurons and PC12 cells. ONOOH-treated Hsp90 induced death in ∼40% of PC12 cells and 60% of motor neurons	[167]
Young adult (4-5 m), middle-aged (10 and 16 m) and old (26-28 m) Fisher 344 male rats	WB, HPLC-MS	SERCA2a nitrated at Tyr294 and Tyr295	Age-dependent nitration and loss of function of the rat skeletal-muscle SR Ca^2+^-ATPase isoforms SERCA1 and SERCA2a	[168]
18 male F344 rats were 7–11 m old (young adult), 22–25 m old (old), and 27–30 m old (very old)	WB	SERCA2a, aconitase, β-enolase, carbonic anhydrase III, triosephosphate isomerase	Significant age-associated increase in nitrotyrosine-modified proteins	[169]
Male F344 BN/F1 rats aged 5, 22, and 34 m	WB, MS/MS	LDL receptor related protein 2, CNP and others	Age-dependent accumulation of nitrated proteins	[170]
Young (4 m) and old (24 m) Fisher344 rats and young (6 m) and old (34 m) Fisher 344 /BN F1 rats	WB, MALDI-TOF MS	α-Fructose aldolase, triosephosphate isomerase, GAPDH and others	Nitrated proteins accumulate at a faster rate in old compared to young tissue, Nitrated proteins are subject to proteasomal degradation, Proteasomal activity declines with increasing age	[171]
17 Fisher 344/BN F1 rats (10-34 m old and 7- 5 m old)	WB, MS/MS	Tropomyosin 1 - α isoform, neurofibromin, cadherin EGF-LAG, seven pass G type receptor 2	Nitrated proteins present in cardiac tissue, their abundance increases with age, 1.5 to 2 fold increase in protein nitration in 34-m vs 5-m old animals	[172]
Young (4-6 m old) and aged (24-26 m old) male C57BL/6 mice	WB, MALDI TOF-MS	Profilin 1, polymerase I, Transcript release factor, peroxiredoxin 6, and others	Significant modification of vascular endothelial cytoskeleton, which potentially contributes to barrier dysfunction, increased vascular permeability and pulmonary oedema	[173]

CNP, 2,3-cyclic nucleotide 3-phosphodiesterase; MS, mass spectrometry; WB, Western blotting

### Methods used to measure the level of nitrated proteins

Among the many technologies available, the most effective and dependable method for the quantification of 3-NT are gas chromatography-mass spectrometry (GC–MS/MS) and liquid chromatography-mass spectrometry (LC–MS/MS). GC–MS/MS and LC–MS/MS based methods showed that the concentration of 3-NT in human plasma is on the threshold of the picomolar (pM) to nanomolar (nM) range and changes only very little upon disease or intervention. These important findings are suitable to serve as the gold standard and as a measure to test the reliability of alternative techniques, such as GC–MS, high performance liquid chromatography (HPLC) with electrochemical detection, or immunological assays. The various antibody assays also need to be validated by these GC–MS/MS or LC–MS/MS methods [141]. Quantitative MS-based analysis is essential for the elucidation of the stoichiometry of the specific tau Lys-directed posttranslation protein modifications that correlate with AD neuropathology. Multiple Reaction Monitoring (MRM) is a targeted mass spectrometry (MS)-based technology that is becoming increasingly utilized for protein quantification. MRM-based approaches have been used to determine the relative abundance of tau polyubiquitylation in human AD brain and global tau in human CSF. In contrast to MS-based discovery proteomics experiments, MRM entails the targeted, simultaneous measurements of peptides that serve as surrogates for the protein targets of interest. MRM-based assays are considered to be the “gold standard” for MS-based targeted protein quantification since they are highly specific, precise, and accurate, and they can be multiplexed (hundreds of peptides can be quantified in a single assay), standardized and readily reproduced. A targeted proteomics method that is similar to MRM is parallel reaction monitoring (PRM) wherein an accurate mass and high-resolution mass spectrometer is used to permit the parallel detection of all target product ions [142].

## CHLORINATIVE PROTEIN MODIFICATIONS IN AGING AND AGE-RELATED NEURODEGERATIVE DISEASES

Chlorinative stress undoubtedly contributes to the pathogenesis of neurodegenerative diseases [143]. In brain, chloride ions are present at the concentration of 10^-2^ – 10^-1^ M [144]. HOCl can be generated with the activation of microglia and myeloperoxidase secretion [145–148]. Moreover, infiltration of monocyte/macrophage and neuronal expression of myeloperoxidase also contribute to the formation of HOCl [149,150]. Supposedly, the brain has poor defence system against HOCl [79,151,152]. Thus, the toxicity of HOCl towards central nervous system tissue was shown [153–155]. Furthermore, MPO was reported to be expressed with increased levels in the cerebral tissue of patients affected by AD [156] and 3-chlorotyrosine as a biomarker of HOCl production was detected in proteins from AD hippocampus. The level of 3-chlorotyrosine in the samples from diseased brain was three-fold higher compared to control samples [150]. Halogenation has a clear effect on the self-assembly of the amyloid β peptide aggregates [157]. However, it can be concluded that a role of protein chlorination in neurodegenerative diseases is not analysed completely yet.

## ADVANCED PROTEIN OXIDATION PRODUCTS (AOPP) IN AGING AND AGE-RELATED DISEASE

A special class of protein modification products, consisting of oxidized, dityrosine-containing, crosslinked proteins formed mainly by reactions of RXS with plasma proteins, predominantly albumin, are so-called advanced oxidation protein products (AOPP). *In vivo*, the generation of chlorinated oxidants is a feature of phagocytic cells containing MPO [158,159]. Witko-Sarsat *et al*. [160] first reported elevated plasma level of AOPPs in uremic patients. High levels of AOPPs were detected in patients on maintenance hemodialysis, followed by those on peritoneal dialysis. Patients with advanced chronic renal failure not yet on dialysis had almost three times higher AOPP levels than healthy subjects.

Size exclusion chromatography of uremic plasma has isolated high-molecular-weight (600 kDa) and low-molecular-weight (80 kDa) AOPPs. The high molecular-weight AOPPs were mostly formed of albumin aggregates, likely resulting from disulfide bridges and/or dityrosine crosslinking. The low molecular weight of AOPPs contained albumin in the monomeric form [160].

Exemplary values of AOPP obtained in studies of aging and neurodegenerative diseases are reported in Table 6.

**Table 6:** Chosen results of studies on the effect of aging and neurodegenerative diseases on the AOPP level in blood serum or plasma.

Subjects studied	AOPP level	Reference
Alzheimer disease	Increased (106.5±27.3 vs 87.5±37.8 µM)	[161]
Chronic schizophrenia	Increased (211.2±159.4 vs 191.7± 146.3 µM)	[162]
Parkinson disease	Increased (65.6 vs 45.6 µM)	[163]
Postmenopausal vs premenopausal women	Increased (118.6±59.1 vs 61.6 ± 16.4 μM)	[164]
Systemic sclerosis	Increased (109.1 vs 75.5 µM)	[165]
Rats, 9-m old (adult) vs 3-m old (young)	Increased (8.3±2.7 vs 6.8±2.3 µM)	[166]
Rats, 22-m old (old) vs 3-m old (young)	Increased (16.1± 4.8 vs 6.8±2.3 µM)	[167]
Rats, 22-m old vs 2-m old	Increased (198.5±44.9 vs 129.3±27.2 µM)	[168]

AOPPs were first recognized as markers of oxidative stress. However, it was reported that AOPPs can also promote ROS production, which leads to a vicious circle. AOPPs activate NADPH oxidase via the protein kinase C-dependent pathway inducing an excessive generation of intracellular superoxide in various renal cells (podocytes, endothelial cells, mesangial cells, and tubular epithelial cells) [169].

AOPPs are assayed spectrophotometrically at 340 nm after treatment samples with KI [160,170]. A kinetic AOPP assay has been proposed [171], but a limited correlation was found to exist between results obtained by the classical and kinetic assay [172].

## BRAIN PROTEIN MODIFICATIONS BY 4-HYDROXY-2,3-*TRANS*-NONENAL IN AGING AND NEURODEGENERATIVE DISEASES

**Figure 2:**
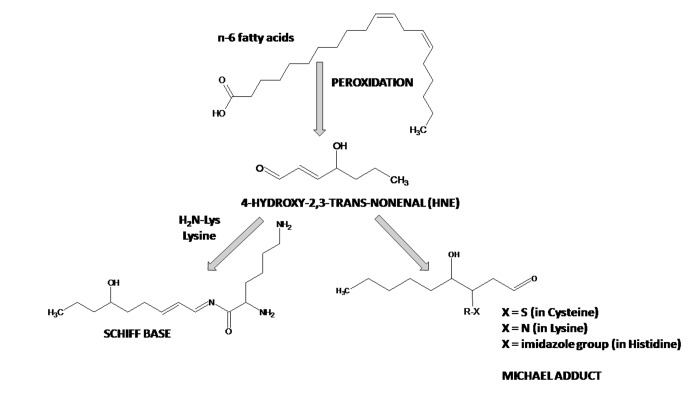
Reactions of 4-hydroxy-2,3-*trans*-nonenal (4-HNE) with proteins.

Post-mitotic neurons are notably vulnerable to lipid peroxidation since the brain has high levels of polyunsaturated fatty acids, high levels of redox transition metal ions, high oxygen consumption, relatively low levels of low-molecular weight antioxidants and antioxidant enzymes. Peroxidation of polyunsaturated fatty acids, especially linoleic acid, linolenic acid and arachidonic acid by non-enzymatic processes leads to the formation of aldehydes, among them 4-HNE is present at very low concentration in plasma, in the range of 0.28–0.68 μM under physiologic conditions, but its concentration in cells, where it is produced, may be higher (≤5 μM) [173]. 4-HNE concentration can be increased as much as by 100 times under OS conditions [174]. Esterbauer's group demonstrated that 4-HNE formation from arachidonic acid is greater in the presence of NADPH-dependent microsomal enzymes [175]. 4-HNE possesses three reactive functions: a C2=C3 double bond, a C1=O carbonyl group and a hydroxyl group on C4. These functions make this electrophilic molecule highly reactive toward nucleophilic thiol and amino groups. 4-HNE can enter the reaction of Michael addition to thiol or amino groups, which involves the C3 of the C2=C3 double bond or can form Schiff bases between the C1 carbonyl group and primary amines. The kinetics of the Schiff base formation is slow and reversible, making Michael-adducts predominant adducts of 4-HNE to proteins. 4-HNE reacts mainly His, Cys and Lys residues in proteins [176,177] (Fig. 1F, Fig. 2). The formation of the 4-HNE-protein adducts is a bioactive marker of pathophysiological processes [178–180]. 4-HNE forms Michael adducts with enzyme peptidylprolyl cis/trans-isomerase A1 (Pin1), which catalyzes conversions of phosphoserine and phosphothreonine-proline from *cis* to *trans* conformation. These adducts were detected by matrix-assisted laser desorption ionization/time-of-flight/time-of-flight (MALDI-TOF/TOF) mass spectrometry at the active site residues His157 and Cys113, with Cys113 being the primary site of 4-HNE modification [181–185]. Protein modifications by 4-HNE impairs glutamate and glucose transport, disrupts Ca^2+^ homeostasis, damages cholinergic neurons thus impairing visuospatial memory and induces apoptosis in PC12 cells (cell line derived from a pheochromocytoma of the rat adrenal medulla) and cultured rat hippocampal neurons [186–188]. Nam et al. (2014) compared N-methyl-D-aspartate receptor type 1 (NMDAR1) and 4-HNE in the hippocampus of D-galactose (D-gal)-induced and naturally aging models of mice [189]. These authors observed an age-dependent reduction of NMDAR1 and an increase in 4-HNE in the dentate gyrus, CA1 and CA3 regions of the hippocampus *via* immunohistochemistry and Western blot analyses. In the D-gal-induced chemical aging model they noted similar changes in NMDAR1 and 4-HNE although the degree of reduction/increase in NMDAR1/HNE was not as severe as that in the naturally aged mice.

4-HNE-protein adducts were found to be elevated in brain tissues and body fluids of AD, PD, Huntington disease as well as ALS subjects [190,191]. 4-HNE-His adducts were reactive with Aβ core of sensile plaques and neurofibrillary tangles [179]. Hardas et al. (2013) detected oxidative modification of lipoic acid, a key co-factor for a number of proteins including pyruvate dehydrogenase and α-ketoglutarate dehydrogenase, by 4-HNE in AD brain [192]. In another study, 4-HNE-Lys adducts were increased in neurons containing neurofibrillary tangles, but also in pyramidal neurons located in the hippocampal tissue sections in AD [193]. The formation 4-HNE adducts with the neuronal glucose transporter GLUT3 and the mitochondrial ATP synthase α subunit in AD brain leads to reduced glucose utilization and energy production in AD [194,195]. Studies conducted by Sultana et al. suggest that 4-HNE-modification of α-enolase, heme oxygenase 1, Collapsin Response Mediator Protein-2 and ATP synthase subunit α are critical in the progression of AD [196]. These authors hypothesized that 4-HNE modification can be not a random event, but occurs on specific proteins, which, in turn, display altered functions. The formation of 4-HNE adducts with α-enolase could inhibit the conversion of plasminogen to plasmin and the degradation of Aβ. In AD brains, the increase of OS leads also to increases of Nrf2 activity as well as, consequently, increases of heme oxygenase 1 level. Heme oxygenase 1 catalyzes the degradation of heme and represents the rate-limiting enzyme in bilirubin production [197]. Collapsin Response Mediator Protein-2 (dihydropyrimidinase-related protein-2) plays an important role in cytoskeletal organization, axonogenesis, axon outgrowth, membrane trafficking and neuronal polarity [198]. The oxidative modification of Collapsin Response Mediator Protein-2, such as formation adducts with 4-HNE, can play an important role in shortening of axons as well as loss of synapses in AD. ATP synthase subunit α, a part of complex V responsible for mitochondrial-resident ATP synthesis. ATP synthase α might by modified by 4-HNE in AD brain, which causes the reduced activity of ATP synthase and reduced ATP levels in AD brain compared to age-matched controls [196]. According to recent study, klotho gene therapy in senescence-accelerated mouse prone-8 (SAMP8) reduced memory deficits, neuronal loss, synaptic damage and 4-HNE levels, and increased mitochondrial SOD-2 and catalase expression. Additionally, the up-regulation of klotho expression decreased Akt and Forkhead box class O1 (FoxO1) phosphorylation. The role of 4-HNE adducts in ALS progression has been recently reviewed by Zarkovic group [180]. ALS is a progressive neurodegenerative disorder characterized by weakness and spasticity, caused by the loss of lower and of upper motor neurons and by secondary neurogenic amyotrophy of striated muscles. An *in vitro* study demonstrated that 4-HNE impairs the glutamate and glucose transport and the choline acetyltransferase activity in cultured motor neurons [199], while human autopsy materials have shown increased levels of 4-HNE, which modifies astrocytic glutamate transporter EAAT2 (excitatory amino acid transporter 2) impairing glutamate transport in ALS. Moreover, 4-HNE is able to target SOD1 in ALS [200]. Kabuta et al. (2015) reported that TDP-43, a major component of ubiquitin-positive inclusions in ALS, is bound by 4-HNE, therefore inducing both proteins into toxic aggregates [201].

It should be mentioned that 4-HNE has also crucial role in αSyn-induced cytotoxicity and neuro-inflammation [202]. These aldehydes can also promote the formation of αSyn oligomers with defined structural properties. Although, 4-HNE modifies αSyn immediately, primarily the His50 residue, oligomer formation only occurs with prolonged incubation times (> 24 h) and involving fewer cross-linking events. 4-HNE can bind to αSyn at an acidic pH, but these modifications cannot promote oligomerization even with increased incubation times [203]. The current objective of research in the field of contribution of 4-HNE-protein adducts is characterization the interactions of 4-HNE with redox sensitive cell signalling proteins. 4-HNE is involved in aging-related signaling pathways, such as NF-κB, AKT, Nrf2 and mTOR. Other signaling pathways involved in aging, for example related to growth factor signaling EGFR, PDGFR and others are also modified by 4-HNE. Understanding how modulation of activities of these signaling pathways contributes to physiological aging and neurodegenerative diseases may pave the way for new therapeutic strategies.

### Assay of 4-HNE-protein adducts

The gold standard in studies of protein modifications by lipid peroxidation products, including 4-HNE, in proteomic studies is mass spectrometry, e. g. matrix-assisted laser desorption ionization/time-of-flight/time-of-flight (MALDI-TOF/TOF), ESI-MS or LC-ESI-CID-MS/MS [204–206]. Antibodies against the His adduct of 4-HNE has allowed for facile detection and quantification of 4-HNE-modified proteins by immunochemical techniques (immunoblotting, immunocytochemistry, immunohistochemistry and immuno-electron microscopy.

Two variants of the 4-HNE-ELISA assay have been developed, both of which are based on the 4-HNE-His monoclonal antibodies. The differences between these two assays concern the analytical protocols and the albumin-HNE standards used, allowing very sensitive determination of low amounts of the 4-HNE-protein adducts (the assay denoted *HNE-His ELISA Fine*) even below 0.025 nmol 4-HNE-His/mg of protein and the one able to detect higher amounts, above 1.5 nmol 4-HNE-His/ mg of protein (the assay denoted *HNE-His ELISA Stress*) [207].

## ROLE OF OXITATIVE STRESS IN THE BLOOD BRAIN BARRIER AGING

The blood brain barrier (BBB) separates the brain and blood with a large surface area (between 12 and 18 m^2^ in the average human adult) [208,209]. The opposing membranes of endothelial cells are connected by tight junctions, which are formed through an intricate network of interacting proteins such as claudins, occludin, junctional adhesion molecules and cytoplasmic proteins [210]. Nitta et al. (2003) demonstrated that claudin-5 is a critical determinant of BBB permeability [211]. In the process of healthy aging an increased “leakage” of BBB may occur, not only due to alteration of thickness of basal lamina, endothelial cells, morphology of pericytes and astrocytes, but also as a result of the changes in expression of transporter proteins at the endothelial cell layer of BBB [212]. Bors et al. (2018) reported that the number of tight junctions decreases, the thickness of basal lamina increases as well as the size of astrocyte endfeet extends with advanced age. These authors also demonstrated that the function of P-glycoprotein 1 (P-gp, ABCB1 Abcb1a/Mdr1a), the most important efflux transporter located on the luminal surface of brain capillary endothelial cells is reduced in old Wistar rats [213]. Reduced BBB expression of P-gp was associated with increased brain parenchymal Aβ40 and Aβ42 levels in aged rats [214], in agreement with the idea that P-gp is an important efflux transporter to remove Aβ from the CNS [215]. Pan et al. (2018) showed that low density lipoprotein receptor-related protein 1 (LRP-1) expression declines with age, which may contribute to Aβ accumulation [209]. Van Assema et al. (2012) studied *in vivo* effects of gender and aging on human BBB P-gp function in a large sample size using PET and (R)-[^11^C]verapamil. These authors reported that decreased BBB P-gp is found with aging; nevertheless, effects of age on BBB P-gp function differ between men and women [216].

The function of BBB can be impaired by ROS/RNS, and these effects are partly mediated by products of lipid peroxidation [217]. The major secondary lipid peroxidation product, 4-HNE can impair the BBB function *via* the decrease of GSH [218]. Wang et al. (2012) reported that overexpression of actin-depolymerizing factor (ADF) blocks the oxidized low-density lipoprotein (ox-LDL)-induced disruption of endothelial barrier. Furthermore, siRNA-mediated downregulation of ADF expression aggravated ox-LDL-induced disruption of endothelial barrier and ROS formation. ADF seems to be a key signaling molecule in the regulation of BBB integrity and suggest that ADF might be used as a target to modulate diseases accompanied by ox-LDL-induced BBB compromise [219]. It should be also mentioned that several studies suggest a link between synucleinopathies and the cholesterol metabolite 27-hydroxycholesterol (27-OHC). 27-OHC is the major cholesterol metabolite in the blood that crosses BBB, and its levels can increase following hypercholesterolemia, aging and OS, which are all factors for increased synucleinopathy risk. 27-OHC can increase αSyn levels and causes the inhibition of the proteasomal function and reduction in heat shock protein 70 levels as potential cellular mechanisms involved in regulation of αSyn [220].

## REMOVAL OF MODIFIED PROTEINS

The level of posttranslationally modified proteins is a resultant of the rate of protein modification and rate of removal of modified proteins. Aging, as well as several age-related diseases are associated with a decreased ability to maintain proteostasis [221]. All cells have a number of quality control mechanisms in order to maintain the stability and functionality of their proteome. The proteostasis network includes both protein stabilization mechanisms (major heat shock proteins) and protein degradation systems (proteasome and lysosome) [222–224]. In addition, there are several modulators of proteotoxicity (like MOAG-4), that operate through distinct pathways [42]. All these systems work in concert to restore the structure of denatured proteins or to promote their degradation, thus preventing the accumulation of damaged components and ensuring the continuous renewal of the intracellular polypeptides. Many studies have shown that aging is accompanied by failure of proteostasis [225], while chronic exposure to denatured or aggregated proteins contributes to the development of age-related neurodegenerative diseases such as AD and PD [221,226].

### The proteasome

The proteasome is a fundamental multicatalytic enzyme complex, which facilitates the degradation of normal as well as abnormal, damaged, denatured and redundant cellular proteins. Proteasomes are located in different cellular compartments (cytoplasm, nucleus and endoplasmic reticulum) and represent approximately up to 1% of the total cellular protein content. The central role of the proteasomes is demonstrated by their participation in numerous and diverse cellular functions, including the regulation of transcription factor abundance, cell cycle and cellular differentiation. The main proteasomal complex is the 30S/26S proteasome and is composed by the 20S catalytic "core" and the 19S regulatory "cap" (summarized in [227]).

The 20S proteasome is a barrel-like structure composed of 28 protein subunits that form a complex of 700 kDa. The two outer rings comprise seven different α subunits, while the interior rings consist of seven β subunits, creating an α1-7/β1-7/β1-7/α1-7 layout. The external α rings control the entry of proteasome’s substrates into the β rings, the site of the proteolytic activity. The α-subunits are additionally responsible for the binding of different factors that regulate the activity and specificity of the catalytic core. Three of the seven β subunits, namely β1, β2 and β5, are proteolytically active, having different substrate specificity. Specifically, β1 has a caspase-like activity (CL or PGPH), β2 a trypsin-like (TL) and β5 a chymotrypsin-like activity (CT-L). The protein hydrolysis occurs after acidic peptide bonds, basic amino acids and hydrophobic amino acids, respectively [228].

The 19S regulatory complex is composed of 19 different subunits that form two heteromeric rings, known as "base" and "lid" [182]. It is responsible for binding, deubiquitination and translocation of the protein substrate in the 20S core. The base is composed of nine subunits, 6 of which (Rpt1-6) possess ATPase activity [230]. Rpn1, Rpn2 and Rpn13 are 3 non-ATPases that are necessary for the proper function of the 19S complex. In addition, since they act as polyubiquitin receptors, these subunits are responsible for the recognition of the ubiquitinated protein substrate [231]. The "lid" bridges the gap between the 20S and the 19S proteasomal particles. This structure is evolutionary conserved and consists of nine RPN subunits (Rpn3, 5 -9, 11, 12 and 15). The "lid" is very flexible structure, necessary for the positioning and the deubiquination of the substrate by the deubiquitinating subunit Rpn11 [232]. Thus, the 19S regulatory complex acts as a very versatile device, which facilitates the access of the protein substrate to the core of the 20S proteasome in an ATP-dependent manner.

**Figure 3:**
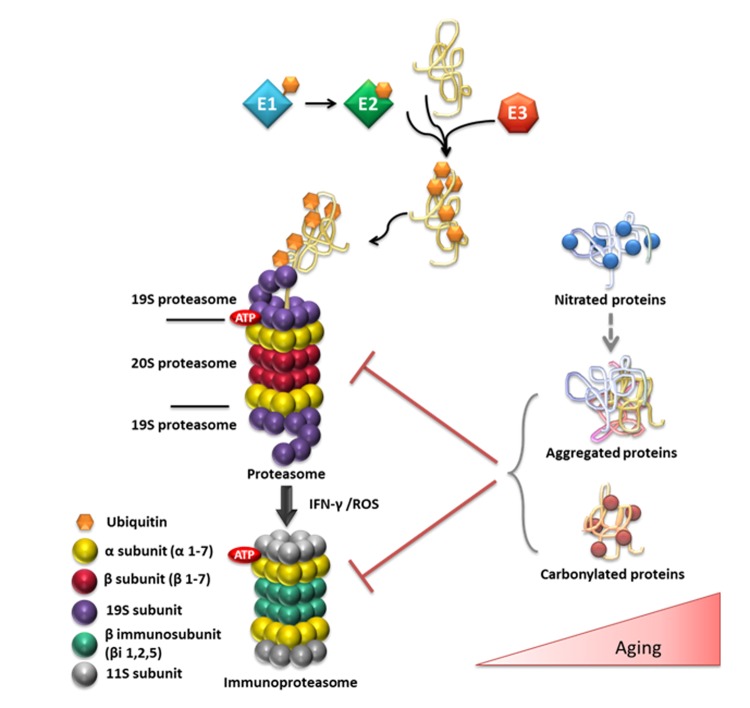
**Overview of the ubiquitin (Ub)/proteasome system and its substrates in relation to aging.** Ub conjugation is mediated by a series of enzymes. The Ub-activating enzyme E1 transfers Ub to the active site of the E2 Ub-conjugating enzyme and the E3 Ub-ligase ligate Ub to the target protein. The ubiquitinated protein is targeted to the 26S proteasome for degradation. The 26S proteasome consists of the 20S catalytic core and of one or two 19S regulatory particles. The 20S proteasome consists of 28 subunits that are divided to two outer α and two central β rings. The immunoproteasome is induced in response to the immunomodulatory cytokine interferon-gamma (IFN-gamma) or in response to the increased OS that is observed during aging. The age-related elevation of OS also causes oxidative damage to proteins, such as carbonylation. In addition, the excessive •NO production during aging can lead to aberrant S-nitrosylation/tyrosine nitration. Nitrated proteins are prone to aggregation and may contribute to the onset and progression of various neurodegenerative diseases, including AD or PD. The accumulation of aggregated or carbonylated proteins inhibit proteasomal activity contributing the observed proteasomal dysfunction during aging and to the advancement of age-related pathologies.

The 26S/30S proteasome is formed by the 20S catalytic core and the 19S regulatory particle. One or two regulatory complexes may bind on the catalytic core, forming the 26S or the 30S complexes, respectively. The substrates of the 26S proteasome are identified by labeling with multiple ubiquitin molecules. The ubiquitin is attached via a three-step procedure, which requires the action of E1 (ubiquitin activation), E2 (ubiquitin conjugation) and E3 (ubiquitin ligase) ligases. Polymeric ubiquitin chains are produced by the repeated action of the E1, E2 and E3 enzymes. The multi-ubiquitin chains signal the identification of the protein substrate for degradation. Upon recognition of the substrate, the poly-ubiquitin chains are removed by deubiquitinating enzymes (DUBs) [226]. The overall mechanisms of ubiquitination and proteasomal degradation are known as the *ubiquitin-proteasome system* (UPS system) (Fig. 3).

Besides the constitutive proteasomes, there are specific specialized proteasomes, formed when the β1, β2 and β5 catalytic subunits become *de novo* substituted by β1i, β2i and β5i subunits, respectively. These subunits are induced in response to the immunomodulatory cytokine interferon-gamma (IFN-gamma). The immunoproteasomes, as they are termed, besides their main role in antigen presentation, are involved in adaptation to OS and in selective degradation of oxidized proteins during aging, possibly in response to chronic inflammation (as summarized in [226]).

### Proteasome and aging

During aging proteostasis collapses [223], resulting in the accumulation of denatured, aggregated or oxidized proteins, which in turn causes cellular damage and impairment of tissues [233]. The proteasomes, being the main proteolytic cellular system responsible for the elimination of nonfunctional or excessive proteins, hold a pivotal role in aging [234].

Young cells and organisms are characterized by an effective preservation of proteostasis. However, this ability is reduced during normal aging. This is evidenced by the increased accumulation of oxidatively modified proteins in senescent cells and tissues, which is indicative of the impairment of protein quality control and of protein degradation systems. Senescent cells have higher levels of proteins bearing modifications, such as oxidative carbonylation, oxidized Met and glycation. Studies *in vivo* and *in vitro* have shown that both the expression and function of the proteasome are negatively affected by aging. Proteasome dysfunction during aging results not only due to the reduced expression of proteasome subunits and the impaired assembly of proteasomal complexes, but also because of the aggregated proteins that inhibit its function. Specifically, the reduction of proteasome activity during aging has been detected in numerous aged human tissues (muscles, lenses, skin, lymphocytes) or other mammalian tissues/organs such as the heart, muscles, spine, brain, liver, adipose tissue and retina (reviewed in [235]).

The activities of the proteasomes decline in senescent human fibroblasts, as a result of a reduction in expression of β subunits [236]. Moreover, it has been shown that the partial inhibition of the proteasomes in young cells causes a p53-mediated premature senescence [237]. On the other hand, the accumulation of damaged proteinaceous materials such as lipofuscin [238] or of protein aggregates [239] during aging, impairs proteasome function. Furthermore, studies in *D. melanogaster* have shown that the age-related disturbances of the 26S proteasome assembly lead to decreased proteasomal activity [240,241]. Notably the naked mole, which is an extremely long-lived rodent, has high levels of proteasome activity, which may contribute to proteostasis maintenance and consequently to the extremely increased lifespan of these animals [242]. Similarly, fibroblasts derived from healthy centenarians have functional proteasomes, with characteristics similar to those of proteasomes from younger donors [243]. Accordingly, human embryonic stem cells (hESCs), that have an unlimited proliferative capacity, exhibit high proteasome activities, as compared to their differentiated counterparts [244]. Recently, the age-related decline of proteasome content and activities, along with the altered proteasome assembly, has been linked with the senescence-related loss of hMSC stemness [245]. Collectively, these studies demonstrate that aging is tightly connected with failures in biosynthesis, assembly and function of the proteasome.

### Proteasome activation

Proteostasis failure is an important determinant of the aging process and is caused by a progressive decline of the respective defense systems. As such, interventions that promote proteostasis may delay aging and reduce the incidence of age-related diseases [246]. For instance, the activation of epidermal growth factor (EGF) signaling extends longevity in nematodes, by increasing the expression of various components of the ubiquitin-proteasome system [247]. Likewise, the enhancement of proteasome activity by deubiquitination inhibitors or by proteasome activators increases the replicative lifespan of yeast *Saccharomyces cerevisiae* [248]. In addition, the overexpression of the β5 catalytic subunit [228] or of the 19S subunit Rpn6 [249] confers an increased lifespan in *C. elegans*.

Similar approaches for activating proteasomes have also proved successful in mammals. The genetic activation of the proteasome has been achieved by the stable overexpression of the catalytic β5 subunit in the fibroblast cell lines WI-38/T and IMR90 [236]. These transfectants have increased ability to degrade oxidized proteins effectively, improved resistance to OS, while the primary IMR90 cells display a 15-20% prolongation of their lifespan. Similarly, the restoration of normal levels of catalytic proteasome subunits ameliorates the aging phenotype in fibroblasts from elderly donors [250]. Overexpression of β5 also promotes proteolysis and resistance to oxidative stress in human epithelial cells [251] and in promyelocytic leukemia HL60 cells [236]. Similar data have been reported in other cell types using different proteasome subunits. For instance, the overexpression of β6 in human bronchial epithelial Beas2B cells increases the activity of the proteasome and protects against the endoplasmic reticulum (ER) stress induced by cigarette smoke [252]. Moreover, an elevation in expression levels of hUMP1/POMP, a chaperone facilitating proteasome assembly, results in increased proteasome activity and protects the cells from OS [205]. Similarly, an increase of PA28 levels in mouse cardiomyocytes stimulates the degradation of denatured proteins, protecting from heart proteinopathy [254]. Additionally, the overexpression of the regulatory 19S subunit Rpn6/PSMD11 enhances the assembly of 26S proteasome in human embryonic stem cells (hESCs) [243]. Remarkably, it has been recently revealed that overexpression of the β5 proteasome subunit in human Wharton-Jelly derived mesenchymal stem cells (WJ-MSCs) resulted not only in increased proteasome activity and assembly, but also induced the expression of additional 26S proteasome subunits. The enhanced proteasome activity was maintained even after extensive culture, protecting the stem cells form the age-related increase of oxidative damage, as indicated by the reduced levels of ROS and of oxidatively modified proteins. Importantly, proteasome activation doubled the replicative lifespan, improved the expression of the core pluripotency factors and enhanced the differentiation capability towards adipocytes, osteocytes and chondrocytes of both young and senescent WJ-MSCs [245].

As genetic manipulation is nοt always feasible for clinical applications, there has been an effort towards the identification of natural or synthetic proteasome activators with antioxidant and anti-aging properties. Substances that directly induce the activity of the proteasome include pollen [255,256], oleuropein [256], curcumin [258] and the synthetic peptide PAP1 (Proteasome Activating Peptide-1) [259]. A different approach concerns the use of compounds that activate the transcription of proteasomal subunits. It is known that the transcription factor Nrf2 (Nuclear factor (erythroid-derived 2)-like 2) induces the expression of antioxidant enzymes including proteasomal subunits [260]. Treatment with 18α-glycyrrhetinic acid (18α-GA) activates Nrf2, which in turn induces proteasome function and results in an enhancement of lifespan of both human fibroblasts [261] and *C. elegans* nematodes [262]. Likewise, treatment with quercetin increases the CT-L proteasomal activity of human fibroblasts and increases their resistance to OS [263]. Finally, activation of Nrf2 by sulforaphane increases pluripotency and self-renewal capacity of hESCs [264]. The analysis of the role of proteostasis maintenance mechanisms in aging, is essential for the rational design of interventions to improve the quality of human life in old age (‘healthspan’), including the treatment of age-related diseases.

## PERSPECTIVES

Abundant evidence demonstrates accumulation of products of protein modifications by ROS, RNS and RXS during aging of humans and model organisms and enhanced accumulation of such products in age-related diseases. New methods of analysis, based mainly on the MS technique, became available allowing for more precise identification of protein modifications and perhaps introduction of specific disease markers. Elucidation of the role of such modifications in aging-related changes and in the progress of diseases is more difficult. Are they only markers or aging and diseases or play a primary role in their development? There are reasons to not exclude the second possibility as these modifications adversely affect protein functions and interactions. Prospective and intervention studies may be helpful in this respect and may point to the possible role of specific protein modifications as possible early disease markers.
